# Easily Installable Wireless Behavioral Monitoring System with Electric Field Sensor for Ordinary Houses

**DOI:** 10.2174/1874431100802010049

**Published:** 2008-03-27

**Authors:** S Tsukamoto, H Hoshino, T Tamura

**Affiliations:** 1 Division of Life Science and Engineering, School of Science and Engineering, Tokyo Denki University, Japan; 2 Department of Electronic and Computer Engineering, Graduate School of Science and Engineering, Tokyo Denki University, Japan; 3 Department of Medical System Engineering, Graduate School of Engineering, Chiba University, Japan

**Keywords:** behavioral monitoring, wireless, electric field, electric appliance.

## Abstract

This paper describes an indoor behavioral monitoring system for improving the quality of life in ordinary houses. It employs a device that uses weak radio waves for transmitting the obtained data and it is designed such that it can be installed by a user without requiring any technical knowledge or extra constructions. This study focuses on determining the usage statistics of home electric appliances by using an electromagnetic field sensor as a detection device. The usage of the home appliances is determined by measuring the electromagnetic field that can be observed in an area near the appliance. It is assumed that these usage statistics could provide information regarding the indoor behavior of a subject. Since the sensor is not direction sensitive and does not require precise positioning and wiring, it can be easily installed in ordinary houses by the end users. For evaluating the practicability of the sensor unit, several simple tests have been performed. The results indicate that the proposed system could be useful for collecting the usage statistics of home appliances.

## 1 INTRODUCTION

In the wake of an increase in the elderly population and medical costs, preventive and epidemiological medicine plays a crucial role in reducing the costs incurred in medical and care insurance and healthcare. In addition, many people with disabilities may be able to live independently in their houses with the aid of an at-home automated health monitoring system. From the viewpoint of the quality of life (QOL), it is important that these people reside in their own homes.

Consequently, by employing a variety of sensor technologies, many noninvasive indoor behavioral monitoring systems have been developed [1, 7], and a relationship between indoor activity and the health condition of a subject was determined ([7], a detailed study about the relationship is also described in the literature). In addition, a home healthcare network system with the cooperation of medical and paramedical staff was also developed [8]. However, these systems comprise many physical sensors such as magnet and motion sensors and require precise positioning and wiring for installation; therefore, most of these systems are installed by an engineer and the installation task is time-consuming. In reality, such systems will be useful in the event of a sudden illness or emergency and should be installed during or immediately after such a situation. In the meantime, even if the system is useful for ensuring the self-healthcare of a person living alone, a questionnaire administered to subjects indicates that continuous monitoring is not required [9, 10]. Therefore, such a system should be easy to install; furthermore, when the system is no longer required, it should be easily removable [10].

Recent studies have revealed that the physical and mental conditions of elderly people are reflected in their behavior; this behavioral pattern changes when they are unhealthy. Nambu et al. have hypothesized that this irregularity can be evaluated from the information regarding the usage of home electric appliances; further, they have proposed an algorithm for the detection of health conditions from television usage statistics [11]. In addition, RFID based object tracking/activity monitoring systems such as Intel’s *iBracelet* were developed. The system has potential for the easy installation and removal, and it will be cost effective since the RFID tags used in the system is inexpensive. However, the users are requested to wear or carry a RFID reader all the time, and that will be an obstacle since the data may not be obtained when the user forget wearing the reader.

The aim of this study is to develop an indoor activity monitoring system that can be both easily installed and removed by end-users. In this study, we focused on the usage of home electric appliances. The sensor unit developed in this study detects the usage of home electric appliances by measuring the electric field strength surrounding them since the operations of most modern-day electric appliances are based on the electromagnetic phenomena. This system employs a wireless data communication device for transmitting the obtained data such that the sensor unit can be used by simply attaching it to an appliance.

## 2 SYSTEM STRUCTURE

The system developed in this study comprises a data storage terminal and many measuring (sensor) units (technically similar to that built by Mote). In this section, the radio system (radio module, packet structure, communication protocols), sensor circuit (two types of sensor were developed in this study), and the threshold setting and feedback mechanism introduced in the sensor unit are described.

### 2.1 Radio Network

Table **1** lists the specifications of the measuring unit, and Fig. (**1a**,**b**) show the schematic diagram and circuit board of the sensor unit, respectively. The measuring unit comprises a PIC microprocessor and low-power radio data communication module. We employed the weak radio device (CDC-TR02B, Design Circuit Inc.) for transmitting obtained data for its low-cost, ease of obtain, and the flexibility of programming. The microprocessor samples the analog output of the sensor circuit at a sampling resolution of 8 bits. The digitized value is then converted into a 16 bit data by Manchester coding and transmitted to the data storage terminal through the radio module. The flag synchronization method is employed for the radio communication; the data packet comprises a flag, target unit ID, sender unit ID, packet serial number, data, and checksum. The radio network, i.e., data relay path, is automatically constructed and modified when a relay failure occurs. Each measuring unit has information regarding the number of hops (distance) to the storage terminal, and the information is broadcast periodically. If a new measuring unit is added, the unit listens to the hop information for a while and builds a network map. In most cases, the data relay path is selected to be shorter hops to the data storage terminal. In the case of a transmission error (when the sensor unit is unable to receive an acknowledgement (ACK) packet from the server), the unit retransmits the data up to 10 times. If a relay failure occurs, the secondary shorter path will be selected. The unit has a USART (RS232C)-type communication port in order to directly connect it to a personal computer (i.e., the unit can also act as a radio interface for the server, as shown in Fig. (**1b**).

### 2.2 General-Purpose Sensor

In order to determine the usage (on/off state) of home electric appliances, we have focused on their electromagnetic phenomena. Electromagnetic phenomena are utilized in many parts of recent electric appliances such as an electromagnetic cooker, microwave ovens, and motors in washing machines. In addition, most appliances are equipped with a power transducer, which comprises coils (electromagnetically coupled transformers), and an electromagnetic field with a frequency of 50 or 60 Hz (frequency of a commercial electric power system) can be observed around it even if the leakage is extremely small. The strength of the electromagnetic field varies and depends on the power consumption of the appliance, which in turn depends on its usage. Thus, the usage statistics can be obtained by monitoring the strength of the surrounding electric field.

Fig. (**2**) shows the circuit diagram of the electric field sensor. The leaked radio wave from an appliance is extracted by the resonator. For the general-purpose sensor, the resonance frequency is set to 50 Hz. The amplified and rectified signals are then low-pass filtered (LPF) by very low frequency sampling in order to determine whether the television set is on or off. Generally, a comparator circuit is necessary for the on/off distinction. However, because the output voltage of the circuit may be affected by the placement of the unit, the comparator circuit, which requires a constant threshold, was not introduced in this study. The on/off status can be differentiated by the raw sampling of the sensor unit output by using the software of the PIC microprocessor.

### 2.3 Television Sensor

The practice of watching television is always associated with the type of program, and this practice is strongly influenced by the physical and mental conditions of the viewer [11]. Therefore, the inclusion of a television set for monitoring is very important. The usage statistics of the television set may be obtained by using the general-purpose sensor; however, in an area where several appliances are placed very close to each other (e.g., video recorder, audio amplifier, etc.), it is difficult to distinguish whether or not the target appliance is in use. In particular, a video deck may start recording by a timer, an activity that is not undertaken by the viewer. An appliance-specific sensor (a television sensor in this case) is required for such a situation, and it can be developed by tuning the focusing frequency.

The image on a television screen is composed of many horizontal lines (scanning lines). By displaying different images at a certain frame rate, the television displays a moving picture. The number of scanning lines *N* and frame rate *f_V_* in conventional television formats are shown in Table **2**. The horizontal scanning frequency *f_H_* can be calculated as the product of *N* and *f_V_*. In a conventional television set comprising a cathode ray tube (CRT), scanning is performed by driving an electromagnet near the electron gun in the CRT. The electromagnetic activity near the CRT during the operation of the television can be detected by obtaining the leaked radio (electromagnetic) waves from the electromagnet. In this manner, information regarding the usage of a television set may be obtained. Since *f_H_* is almost the same in all the television formats, the resonance frequency is set to 15.7 kHz, which is also the intermediate frequency.

### 2.4 Threshold Setting and Feedback Method

Providing feedback regarding the correct installation of the sensor unit (usage information regarding a target appliance may be obtained incorrectly) is very important for installation by the end users. In this study, a simple threshold decision mechanism that utilizes a microprocessor used in digital data communication is adopted. Two buttons are introduced and connected to the microprocessor; the first is to be pressed when the target appliance is not in use, and the second, when the appliance is in use. The microprocessor samples and memorizes the sensor outputs when one of these buttons is pressed; then, the threshold value is set to the median of these two values. The microprocessor samples the sensor output at 10 Hz, and an LED is powered on when the sampled value exceeds the threshold. By introducing the threshold decision mechanism, the feedback LED will function without the preset threshold value. The end users can confirm whether or not the sensor unit is working correctly by checking the feedback LED and actual status of the target appliance.

## 3 EXPERIMENTS

Several experiments were performed in order to assess the practicability of the developed system. As the first experiment, we evaluated the wireless data communication range by a packet transmission test. The behavior of sensor circuit (relationship between input and output signals) is then confirmed with simple power on-off test. The validity of the threshold setting mechanism and the dependence of sensor output intensity on appliances are also confirmed. As to be the easily installable monitoring system, the installability evaluation was performed, and finally the indoor activity monitoring by the proposed system was demonstrated.

### 3.1 Data Communication Range

A sender (measurement) unit was placed at distances of 0.5 m to 12.5 m from the data storage terminal at intervals of 0.5 m and transmitted 50 packets at each position. The packet receiving success rate was evaluated in this experiment.

In addition, to confirm the availability of the multi-hop system, a data relay unit (which can also be a measuring unit) was placed at a distance of 5.0 m from the data storage terminal, and the abovementioned packet transmission test was performed.

### 3.2 Behavior of Sensor Circuit

To confirm the behavior of the proposed sensor circuit, we observed the obtained raw signal (leaked radio waves from the television set), processed signal (resonance, amplification, and rectification), and filtered sensor outputs. These signals were recorded using Tektronix TDS210. The A/D converter in the PIC microprocessor was not used in this experiment. For the evaluation of the general-purpose sensor, we selected a microwave oven as one of the home appliances.

### 3.3 Appliances Power On/Off Test

The applicability of the sensor was evaluated by a simple power on/off test. A television sensor was used in this experiment. The sensor unit was placed on the target appliance, and the data storage server was placed at a distance of ~2 m. The sensor output was automatically sampled by the PIC microprocessor installed in the sensor unit at a sampling frequency of 10 Hz and sampling resolution of 8 bits. A reference voltage of 5.0 V was used for the A/D converter. The obtained data was then transmitted to the server. A simple handshake protocol was employed in this experiment. In the case of a transmission error (when the sensor unit was unable to receive an ACK packet from the server), the unit retransmitted the data without any limitation on the number of retransmissions.

### 3.4 Behavior of Threshold Setting

**Fig. (1):**
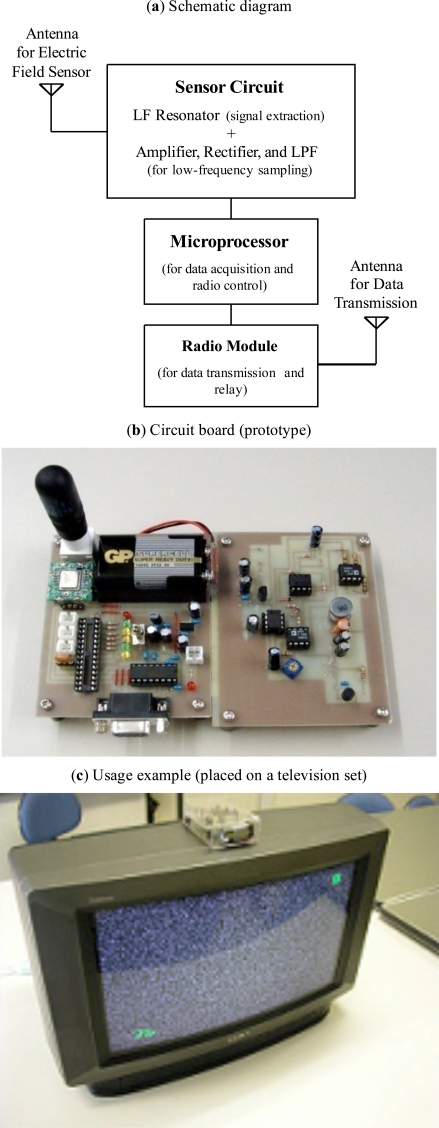
Measuring unit.

In order to confirm the validity of the threshold setting mechanism, a simple evaluation test was performed. A general-purpose sensor (which responds to the frequency of a commercial electric power system) and television set were used in this experiment. The sensor unit was placed on the television set (Fig. **1c**). The initial condition of this experiment was that the television set was not in use (power-off state). In the beginning, the first button was pressed to sample the sensor output (background noise). The television set was then powered on, and the second button was pressed (the microprocessor samples the sensor output and updates the threshold value).

In addition, to ensure the practicability of the sensor unit, the sensor output voltages of 10 domestic home electric appliances such as a microwave oven, television set, and coffee maker were evaluated.

### 3.5 Dependence of Sensor Output on Appliances

The dependence of the sensor output on different appliances was evaluated. Several microwave ovens with radio frequency outputs of 500 W were evaluated with respect to the general-purpose sensor. In the case of the television sensor, we confirmed the dependence of the sensor output on the screen size; several conventional television sets comprising CRTs were evaluated in this study.

### 3.6 Installability by End Users

The proposed system is intended to be installable by the end users; thus, this installability was evaluated. A CRT-type television set and television sensor (which responds to a 15.7 kHz frequency component) were used in this experiment. Both the installation time and sensor output intensity were evaluated.

For comparison purposes, we evaluated the installation time of a current-detector-type sensor unit as an ordinary system. In this experiment, a small adapter was used as the current detector. A television set was placed near a wall socket and the power cable was placed such that it would be clearly visible.

Five healthy volunteers (female, age: 48.0 ± 11.7 years), from whom prior verbal consent was obtained, participated in this experiment. They were requested to place the sensor unit on the television set. In the comparison test, the volunteers were requested to connect the adapter between the wall socket and power plug.

### 3.7 Behavioral Monitoring

**Fig. (3):**
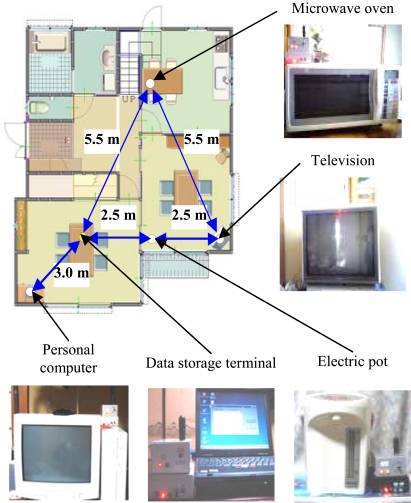
Experimental setup for behavioral monitoring. Four sensor units and one data storage terminal are installed.

To assess the practicability of the proposed system, four sensor units were installed in an ordinary house (Fig. **3**); the target appliances in this experiment were a microwave oven, electric pot, television set, and personal computer. One healthy volunteer (male, age: 23 years), from whom prior verbal consent was obtained, participated in this experiment.

## 4 RESULTS

### 4.1 Data Communication Range

Fig. (**4**) shows the packet transmission success rate at each distance. The results indicate that direct communication range of the measuring unit developed in this study is 6.0 m, and the communication distance can be extended by the multihop (data relay) technology.

### 4.2 Behavior of Sensor Circuit

Fig. (**5**) shows the results of the case when the appliances (i.e., the microwave oven) are not in use. Fig. (**5a**) shows the signal received by the antenna of the sensor circuit; no significant component exists in this signal. A very small component of the power-line frequency of commercial electric power systems can be observed anywhere in the house; however, the gain of the amplifier is not high. Thus, this type of “noise” is ignored. As a result, the filtered output is almost 0 V (approximately 60 mV in the general-purpose sensor).

**Fig. (6):**
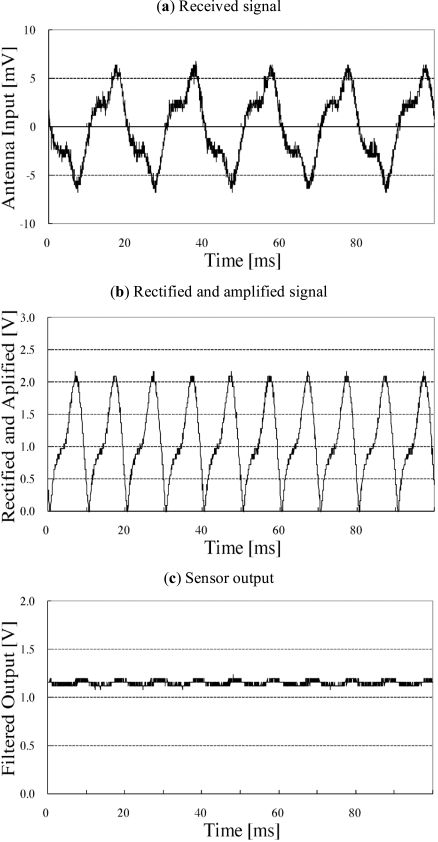
Signal processing of general-purpose sensor (microwave oven). The received signal mainly comprises a 50 Hz component derived from the commercial electric power system.

Fig. (**6**) shows the experimental result obtained by the general-purpose sensor with a microwave oven. Fig. (**6a**) shows the obtained radio wave leakage. The signal mainly comprises 50 Hz waves. Since the radiated waves are not sinusoidal, the microwave oven evaluated in this experiment may also have radiated other frequency components (harmonics). This phenomenon should be thoroughly investigated for the development of a microwave-oven-specialized sensor. The 50 Hz component is extracted by the resonator and subsequently amplified and rectified (Fig. **6b**) shows the results of signal processing). The filtered output (Fig. **6c**) shows that when the microwave oven is in use, the sensor output increases to ~1.18 V.

Fig. (**7a**) shows the signals obtained from a television set by the television sensor when the television set is in use. The signal mainly comprises 15.7 kHz pulses. Fig. (**7b**) shows the results of signal processing. Fig. (**7c**) shows the filtered output of the signal shown in Fig. (**7b**). When the television set is in use, the sensor output increases to ~0.95 V.

### 4.3 Appliances Power On/Off Test

Fig. (**8**) shows the results of the on/off test of the television. During the experiment, on an average, approximately 10 retransmissions and one instance of data corruption occurred for each data transmission. Since the data recorded at the server comprised several duplicate data entries obtained from the sensor unit, most of the retransmissions were assumed to be caused by packet losses. However, based on this result, we can estimate the duration of the use of the television set; thus, the sensor unit is suitable for practical use.

### 4.4 Behavior of Threshold Setting

Fig. (**9**) shows the results of the threshold setting experiment. At the beginning of the experiment, the television set was in the power-off state; in this state, the sensor output was approximately 30 mV, which was environmental noise. In this experiment, the sensor output was approximately 0.1 V even in the power-on state; however, by using the threshold mechanism, the threshold value was set to approximately 0.06 V; therefore, an accurate feedback could be obtained.

The results of the evaluation of 10 electric appliances were as follows: the output voltages of the sensor unit when the appliance was in use and not in use were approximately 1.00 ± 0.28 V and 31 ± 24 mV, respectively. To assess the result statistically, we used the One-way ANOVA method to analyze differences between these two states. Statistically significant *F* values were then followed up with Scheffé’s post-hoc tests. The statistical significance (*p* < 0.01) of these two voltages (by power states) was determined. Therefore, the output voltage of 0.1 V, similar to that obtained in the threshold setting experiment, was observed in a few cases.

However, such cases exist in reality, and in the event that the feedback fails, the end user may not install the unit because it appears to be nonfunctional.

### 4.5 Dependence of Sensor Output on Appliances

Table **3** shows the results of the evaluation of several microwave ovens. There was no tendency based on the experiment; however, the results show that when the microwave oven was not in use, the output voltage was almost 1 V.

Table **4** shows the results of the evaluation of the dependence of the sensor output on the screen size. The average voltage of the sensor output is inversely proportional to the screen size, even though the power consumption of the television set is directly proportional to the screen size. This tendency is attributed to the fact that the radio wave leakage is directly proportional to the power consumption. However, the distance between the source of radio wave leakage and the antenna of the sensor circuit also increases with the cabinet size of the television. As a result, an increase in the distance decreases the intensity of the received signal.

### 4.6 Installability by End Users

The time required by the end users to install the proposed sensor unit was evaluated to be 5.4 ± 0.89 s, whereas that required to install the ordinary device (current detector) was 21.8 ± 7.9 s (the statistical significance was determined to be p < 0.01). In this experiment, the wall socket was easily accessible; however, the questionnaire administered to the subjects indicated that it is difficult for them to access a wall socket in their houses. Therefore, the proposed sensor unit is preferred over the current detector sensor. In most cases, the socket is placed behind the appliances and the subjects do not usually access it.

The output intensities of the sensor circuit when the unit was installed by the end users were 0.764 ± 0.47 V and 0.073 ± 0.01 V in the cases that the television set was powered on and off, respectively (the statistical significance was determined to be *p* = 0.01). This result suggests that the proposed sensor unit will work correctly even if it is installed by the end users.

### 4.7 Behavioral Monitoring

Fig. (**10**) shows the typical experimental result of the installation of the proposed system in an ordinary house. The vertical axes are normalized by the maximum value of the sensor output. The result shows that we can confirm the usage of these four appliances quantitatively. It appears that the subject usually powers on the television after midnight. Further, the subject uses the microwave oven in the morning and evening for preparing meals. In addition, the usage of the electric pot (boiling water) is higher when the television set is powered on. The electric pot may be activated periodically in order to maintain the temperature of hot water; however, such activities indicate that the electric pot is frequently emptied, after which water is poured and boiled. After the experiment, the subject stated that the microwave oven was frequently used for preparing meals, and that he would sleep without turning off the television set. Therefore, we can estimate the indoor activity by considering the summation of the activities of the electric appliances. Furthermore, the daily routine of this subject can be estimated based on the usage statistics of the microwave oven.

## 5 DISCUSSION

The data communication range of the measuring unit developed in this study was approximately 6.0 m. The range may not be extended under the low-power radio regulation law in Japan. However, the 6.0 m range is sufficient for traditional Japanese houses; by placing several sensor units in each room, the usage information can be obtained by the multihop technology. The packet transmission success rates for distances of 9.5, 10.0, and 10.5 m are slightly low, and the reason for this is as follows. The probability of a packet transmission error is higher in the cases of a greater distance (approximately 5.0 m), and the error ratio is doubled due to multihopping. In most cases, the packet losses are caused by dropping the first byte of the packet. This problem may be solved by devising an appropriate structure of the data packet. Further, we are considering changing the radio module to Mote, Zigbee, or some other radio system that employs FSK or PSK for radio transmission in order to address this problem. Introducing KNX/RF (http://www.knx.org) will be one of the solutions. If the KNX system is already installed in a target residence, the monitoring system could be seamlessly integrated into the building system.

The electromagnetic phenomenon is widely used in electronic/electrical machines such as home appliances, which include motors, transformers, and relay switches. Furthermore, an electromagnetic field exists around an electric wire when the connected appliance is in use. A clamp meter focuses on and uses this phenomenon for estimating the amount of electric current in an electric wire. The sensors developed in this study can also be considered as one of the applications of the clamp meter, the differences being that the sensor developed in this study is able to extract a specific frequency of the electric current and there is no need to separate the power cable for the installation (most power cables comprise two or three wires, and the clamp meter should clamp only one of these wires).

As described in section 2.2, the general-purpose sensor responds to the power-line frequency of the commercial electric power system. Thus, the sensor can be adapted to almost all appliances that have a built-in transformer, provided the transformer comprises coils. A simple evaluation has shown that the sensor could be compatible with the following appliances: electric pot (Zojirushi CD-LE40, CD-GS50, maximum power consumption: 985 W), coffee maker (Sanyo, SAC-MST6, 850 W), electric fan (Yamazen, BX-A252, 42 W), humidifier (National, FE-KHA05, 433 W), and notebook computer (IBM X31, Toshiba DynaBook CX/E216L). The sensor can work even when the area around these appliances, in which the electromagnetic signal can be detected, is relatively small. This area may be widened by increasing the amplifier gain; however, such an increase also amplifies the noise resulting from the power lines in the house, as mentioned above and in section 4.2. The introduction of an automatic gain controller (AGC) or variation in the gain is required to solve this problem; on the other hand, a small detectable area may prevent the contamination of the signal by signals from other appliances.

By introducing the threshold decision mechanism, the feedback operation can be performed in the case of appliances with low power consumption (i.e., small detectable area). The threshold setting is somewhat bothersome for the end users; however, it enables appropriate feedback. However, even if the threshold was set by end-users, it will not adapt to the movement of the sensor unit that will occur during cleaning or earthquake etc., and this could be a limitation of the threshold setting mechanism.

The experimental results reveal that the proposed system can be installed by the end users in a short time, and the usage information is accurately obtained without the dependence of the sensor output intensity on a specific electric appliance. The sensors developed in this study adapt to almost all home appliances.

The proposed system can be considered as a part of an ordinary system (watt meter in the system of [7]). Especially, the usage information regarding television set obtained by the proposed system is almost same to the one obtained by the traditional current detector, such as [6]. If we focus on the starting point of using each appliance, the analysis method proposed in the literature [11] may be applied. The important point of this study is that the easily installable monitoring system was developed, and this will be very important to find solid evidence.

In a long term evaluation we need to refer this system with the medical record with medical appliance and /or physiological data in the epidemiological point of view and to prove the relationship between medical record and physical results from our system. Nevertheless, we definitely convince that this system can be applied for the home health care and be useful an early diagnosis from previous and our studies.

## 6 CONCLUSIONS

In this study, we have developed a behavioral monitoring system for ordinary houses that focuses on the activity of home electric appliances; further, the electric field strength surrounding them has been developed; the system fulfils the requirements of simple installation and removal. The experimental results reveal that the proposed system can be easily installed by the end users (installed in a short time), and their behavioral information can be accurately obtained. Increasing the installability of the monitoring system will be useful not only for temporal construction of such a monitoring system into an ordinary house but also for increasing the number of experiment; this will enable us to find firmer evidence in the future. Further clinical evaluations are left for our future work.

## Figures and Tables

**Fig. (2):**
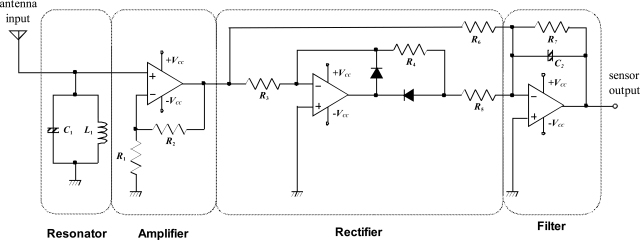
Circuit diagram of electric field sensor.

**Fig. (4):**
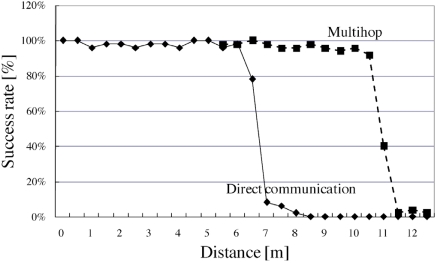
Radio communication range. A sender unit transmits 50 packets at each distance from the data storage terminal, and packet communication success rate is calculated.

**Fig. (5):**
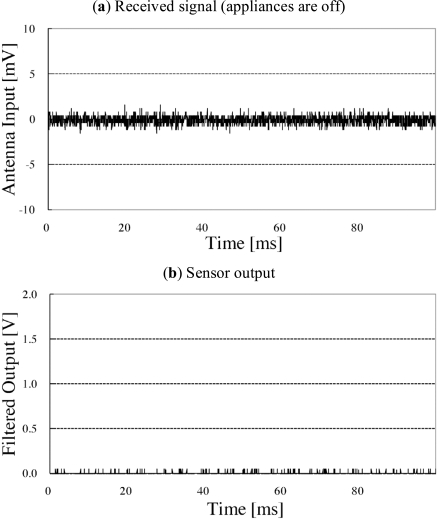
Environmental noise and sensor output.

**Fig. (7):**
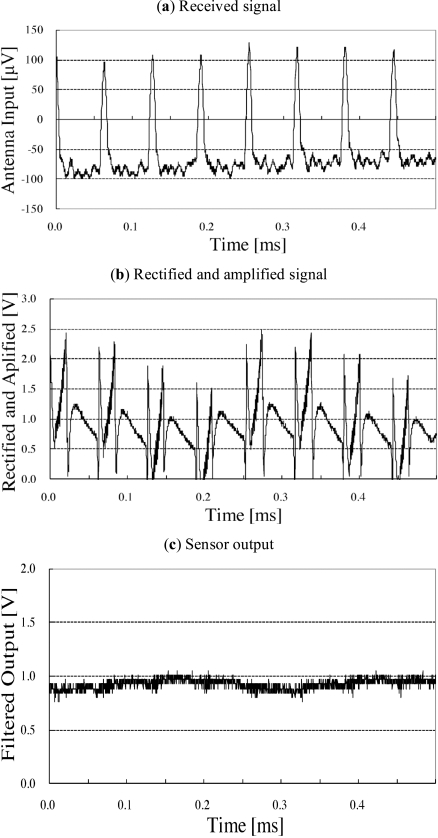
Signal processing of television sensor. Received signal mainly consists of 15.7 kHz pulses which come from electromagnet for horizontal scanning of CRT.

**Fig. (8):**
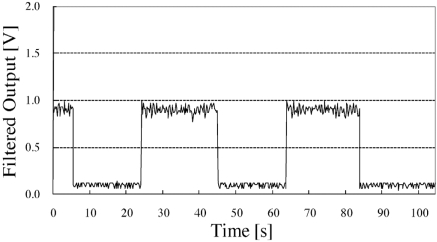
Experimental result of television on/off test.

**Fig. (9):**
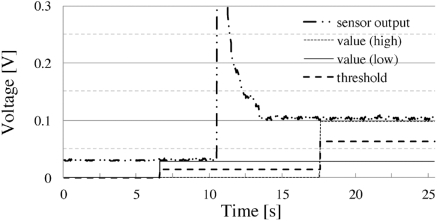
Time-course of setting the threshold. At the beginning of the experiment, the target appliance is in the power-off state. By pressing the first button, the sensor output (noise) is recorded. The appliance is then powered on and the threshold value is adjusted by pressing the second button.

**Fig. (10):**
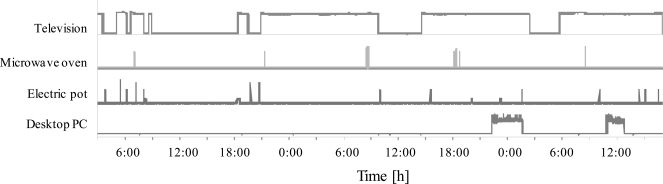
Experimental results of the proposed system (electric appliance usage monitoring).

**Table 1:** Specifications of the Measuring Unit

Item	Specification
**Processor**	PIC16F876 (10 MHz, Microchip Technology Inc.)
**Radio module**	CDC-TR02B (315 MHz, 115.2 kbps, amplitude shift keying, Design Circuit Inc.)
**Antenna**	RH-3 (1/4-λ, Diamond)
**Network structure**	Ad-hoc (CSMA)
**Target (resonance) frequency**	50/60 Hz for general-purpose sensor, 15.7 kHz for television
**Unit size (H × W × D)**	100 × 95 × 120 mm

**Table 2:** Definitions of Conventional Television Formats

Item	Format	
	NTSC, PAL-M, PAL 60	PAL, SECAM
**Scanning line *N *[lines]**	525	625
**Frame rate *f_V_* [Hz]**	29.97	25
**Horizontal scanning frequency *f_H_* [kHz]**	15.734	15.625

**Table 3:** Output Voltage of General-Purpose Sensor (Microwave Ovens)

**Year of Manufacture**	1994	1996	1997
**Rated Power Consumption [W]**	960	920	1000
**Average Sensor Output [V]**	0.86	1.18	1.00

**Table 4:** Dependence of Sensor Output (Television Sensor) on Screen Size

Screen Size [in]	14	24	27	33
**Year of Manufacture**	1995	1995	1989	1989
**Rated Power Consumption [W]**	67	131	153	169
**Average Sensor Output [V]**	1.01	1.01	0.97	0.92
